# Deep pelagic food web structure as revealed by *in situ* feeding observations

**DOI:** 10.1098/rspb.2017.2116

**Published:** 2017-12-06

**Authors:** C. Anela Choy, Steven H. D. Haddock, Bruce H. Robison

**Affiliations:** Monterey Bay Aquarium Research Institute, 7700 Sandholdt Road, Moss Landing, CA 95039, USA

**Keywords:** deep sea, marine ecosystems, empirical food web data, remotely operated vehicles, trophodynamics, predator–prey relationships

## Abstract

Food web linkages, or the feeding relationships between species inhabiting a shared ecosystem, are an ecological lens through which ecosystem structure and function can be assessed, and thus are fundamental to informing sustainable resource management. Empirical feeding datasets have traditionally been painstakingly generated from stomach content analysis, direct observations and from biochemical trophic markers (stable isotopes, fatty acids, molecular tools). Each approach carries inherent biases and limitations, as well as advantages. Here, using 27 years (1991–2016) of *in situ* feeding observations collected by remotely operated vehicles (ROVs), we quantitatively characterize the deep pelagic food web of central California within the California Current, complementing existing studies of diet and trophic interactions with a unique perspective. Seven hundred and forty-three independent feeding events were observed with ROVs from near-surface waters down to depths approaching 4000 m, involving an assemblage of 84 different predators and 82 different prey types, for a total of 242 unique feeding relationships. The greatest diversity of prey was consumed by narcomedusae, followed by physonect siphonophores, ctenophores and cephalopods. We highlight key interactions within the poorly understood ‘jelly web’, showing the importance of medusae, ctenophores and siphonophores as key predators, whose ecological significance is comparable to large fish and squid species within the central California deep pelagic food web. Gelatinous predators are often thought to comprise relatively inefficient trophic pathways within marine communities, but we build upon previous findings to document their substantial and integral roles in deep pelagic food webs.

## 1. Introduction

Food webs are networks of feeding interactions that encompass overall energy flow between resources and consumers within a given ecosystem. These interlocking food chains are an established, central foundation of modern ecological investigations [1,2]. The backbone of a food web investigation comprises empirical information about the feeding relationships between individual species or functional groups. Gathering quantitative feeding data for all members of an ecosystem is challenging, if not impossible for vast habitats with high species diversity, and food webs are thus commonly constructed from compiled observations or diet studies often limited in space or time, or by taxonomic resolution [3].

Within the deep sea, Earth's largest ecosystem, the challenge of gathering empirical feeding data for food webs is particularly formidable due to logistical access and sampling constraints [4]. Analysing the contents of a consumer's stomach (gut or stomach content analysis, SCA) is the common and most directly quantitative way of inferring diet, and is an irreplaceable approach for determining the taxonomic identity of food web components. However, for deeper-dwelling fishes with internal gas spaces, stomach eversion can confound this approach [5]. To resolve broad, generalizable feeding relationships SCA often requires larger sample sizes than those that are attainable from vast, highly dynamic deep-sea ecosystems [5]. SCA may also fail to quantify diaphanous or amorphous gelatinous prey that are readily digested and become quickly unrecognizable [6,7]. Likewise, SCA of net-captured animals is substantially biased towards hard-bodied predators, whereas gelatinous carnivores are typically under-represented due to net extrusion. Lastly, SCA of consumers collected by trawling can be compromised by net feeding [8].

Empirical data on trophic links have recently been generated from biochemical tracers like stable isotopes and fatty acids [9–13], which integrate feeding across longer time scales (weeks to months) in contrast to the snapshots of feeding from SCA. These approaches are often limited by their inability to resolve prey taxonomic identities, providing instead, general trophic trends.

Another source of diet data for documenting food webs comes from *in situ* observations of feeding (e.g. [14,15]). Generally, to capture a diversity of feeding, this method requires sustained periods of observation and has been typically limited to shallow-water habitats accessible by snorkel and SCUBA. The growing use of submersibles and remotely operated vehicles (ROVs) in deep-sea habitats has increased the availability and quality of *in situ* feeding observations. However, few efforts have integrated individual observations into the construction of ecosystem-wide food webs that capture the diversity of inhabitants, flexibility in feeding behaviours and their resultant energy flow patterns.

Waters offshore of central California are characterized by marked spring and summer coastal upwelling events, followed by intrusion of oceanic California Current water and then, the northward movement of warm surface water along the coast during winter [16]. The pelagic fauna of this region is a rich, diverse assemblage of plankton, fishes, cephalopods, crustaceans and gelatinous animals, shifting with increasing depth [17]. Across three depth zones—the epipelagic (approx. 0–200 m), mesopelagic (approx. 200–1000 m) and bathypelagic (approx. 1000–4000 m and deeper)—trophic structure can be generalized into four tiers of prey and consumer guilds: phytoplankton, zooplankton, micronekton and higher-order carnivores [4,18]. Little is known about the overall flow of energy through different, interlocking food chains within the food web and how, or if, seemingly disparate communities are connected vertically across stratified habitats. A more detailed understanding of the most important species-specific, predator–prey relationships is required to implement ecosystem-based fishery management [19] and to address the growing need for predictive understanding of how entire ecosystems will respond to climate-induced changes [20,21].

We compile a unique dataset comprising 27 years (1991–2016) of *in situ* deep pelagic feeding observations from within the oceanic ecosystem spanning up to 250 km offshore of the greater Monterey Bay region, collected by the Monterey Bay Aquarium Research Institute (MBARI) using scientifically optimized ROVs across the full water column (0–4000 m). We identify the most commonly observed predator–prey interactions based on quantified observations over the 27-year time series, looking across depth zones and seasons, where possible. We assemble ecosystem-level schematics of overall energy flow and synthesize ecologically distinct primary food web pathways. Some ROV-based observations of trophic links between midwater species in Monterey Bay have been published (e.g. [22–24]). The present effort is the first to consolidate all such observations into a food web synthesis that complements the existing literature based on other approaches, providing new insights into the overall complexity and functioning of open-ocean food webs.

## 2. Material and methods

### (a) Study site and data collection

**Figure 1.**
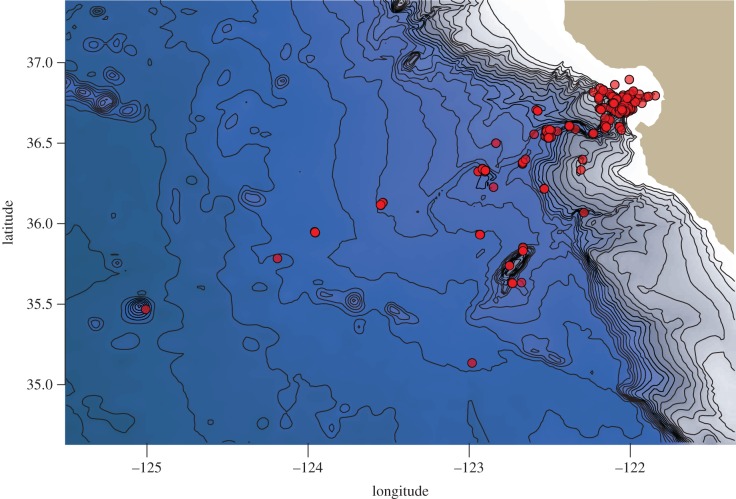
Locations of observed feeding interactions included in this study, made during ROV dives by the Monterey Bay Aquarium Research Institute (MBARI) in the offshore central California ecosystem across the years 1991–2016.

MBARI's ROV programme has been regularly sampling and observing the midwater ecosystem (approx. 50–4000 m) of Monterey Bay and waters offshore since 1989, conducting thousands of ROV dives since the programme's inception. The primary research platforms comprise three ROVs: *Ventana*, which is electro-hydraulic and operates between 50 and 1850 m, *Doc Ricketts*, a newer electro-hydraulic vehicle that operates between 200 and 4000 m, and the now-retired *Tiburon*, an electric vehicle, which operated to 4000 m between 1996 and 2007. All vehicles were fitted with high-definition video cameras and environmental data instrumentation (e.g. depth, temperature, salinity and oxygen sensors). The most frequently visited midwater station since 1989 is a time-series site, Midwater 1 (36.7° N, 122.05° W; 1600 m deep), where many of these feeding interactions were observed. Figure 1 details the locations for all feeding observations included in this study. Owing to operational constraints, the overwhelming majority of these data were collected during daylight hours, and thus we have not explicitly addressed the ecological effects of diel vertical migration on feeding behaviour with this dataset. ROV operations during the fall and winter seasons do, however, overlap with the descent (pre-dawn) and ascent (post-dusk) of the deep-scattering layer.

Feeding interactions include observations of prey within the grasp of a predator's arms, tentacles or mouth, or of prey contained yet visible within the gut of a transparent predator. Prey sizes ranged from millimetres (copepods, radiolarians) to metres (siphonophores). While the physical presence of an ROV has a demonstrated influence on animal behaviour [25], in virtually all of the interactions reported here, prey capture and/or ingestion had already occurred before the arrival of the ROV. Behavioural modifications associated with ROV-based observations will disproportionately impact mobile animals that are optically and/or acoustically sensitive. Thus, a primary bias associated with this dataset includes attraction to and/or avoidance by some fishes, squid and crustaceans, which is a behavioural response not usually evident among gelatinous animals. Consequently, our results are shifted towards gelatinous predators and cephalopods, which generally exhibit little or no avoidance.

### (b) Video annotation of remotely operated vehicle footage

**Figure 2.**
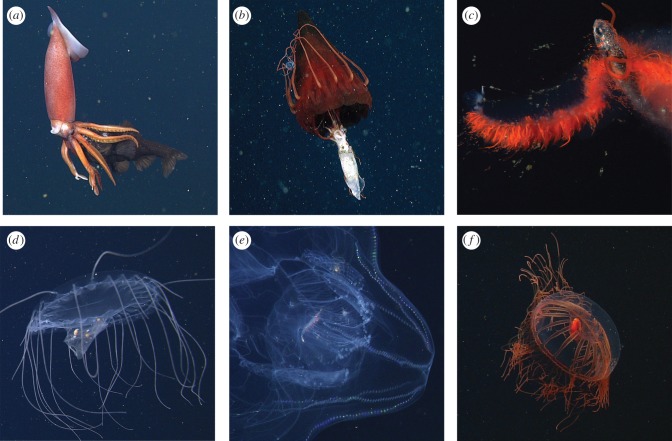
A suite of six illustrative ROV frame grabs of pelagic predators and their prey included in this study. No scale bars for size are available from these sequences. From left to right, top to bottom: (*a*) *Gonatus* sp. (squid) feeding on a bathylagid fish (Bathylagidae); (*b*) *Periphylla periphylla*, the helmet jellyfish, feeding on a gonatid squid (Gonatidae), with a small narcomedusa (*Aegina* sp.) also captured; (*c*) an undescribed physonect siphonophore known as ‘the galaxy siphonophore’ feeding on a lanternfish of the family Myctophidae; (*d*) a narcomedusa, *Solmissus*, ingesting a salp chain (Salpida); (*e*) the ctenophore *Thalassocalyce inconstans*, with a euphausiid (Euphausiacea) in its gut; and (*f*) the trachymedusa, *Halitrephes maasi*, with a large, red mysid (Mysidae) in its gut.

ROV video of feeding events collected during ‘fly by’ (in transit) and ‘parked’ (focused documentation while stopped) modes of observation was annotated in MBARI's Video Annotation Reference System (VARS) [26] by the authors and specialized video research technicians with midwater expertise. Recorded feeding interactions were analysed by identifying organisms to the lowest possible, most specific taxonomic level (i.e. a ‘prey’ concept), and for each annotation predator and prey roles were denoted depending on who was actively ingesting whom. For transparent and translucent animals, visible prey items within a predator's stomach were also identified (figure 2*d,e,f*). Within VARS, environmental and collection data fields (date, depth, latitude, longitude, hydrographic parameters) accompanying each unique feeding interaction were merged for data analysis. Video annotations of feeding interactions were processed to identify unique predator–prey feeding events, based on ROV dive number, depth and tape time code, and were then tabulated according to identified taxonomic levels for both predators and prey.

### (c) Data analysis and filtering

Predator–prey interactions were filtered using custom Python and R scripts to remove feeding events occurring on or near the seafloor (i.e. benthic or benthopelagic). Twenty-seven interactions involving pelagic amphipods (mostly belonging to the suborder Hyperiidea), originally identified as either predator or prey, were excluded. Pelagic amphipods typically maintain symbiotic or parasitic relationships with many gelatinous host species [23,24], and were thus not considered to be ecologically comparable to the rest of the dataset. Unless amphipods were observed to be actively ingesting a prey item, they were excluded. Lastly, we spatially define the study ecosystem as waters between 35–38° N and 121–126° W, a maximum distance of approximately 275 km from the nearest shore.

**Figure 3.**
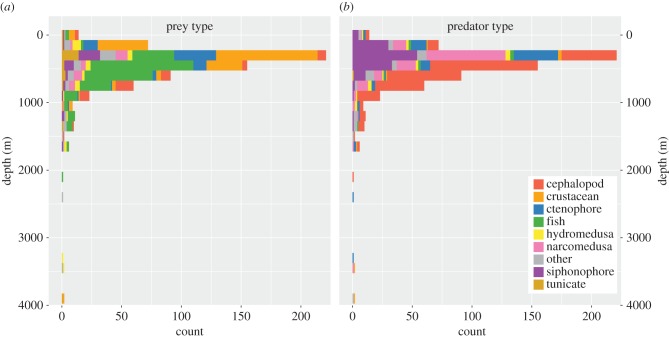
Counts and depth distributions of 718 pelagic feeding observations categorized into nine different broad animal groupings, made by ROVs within the study ecosystem between the years 1991 and 2016. (*a*) Prey and (*b*) predator identities and depth distributions illustrate the depth distributions and general animal identities of the feeding interactions presented throughout this manuscript.

**Figure 4.**
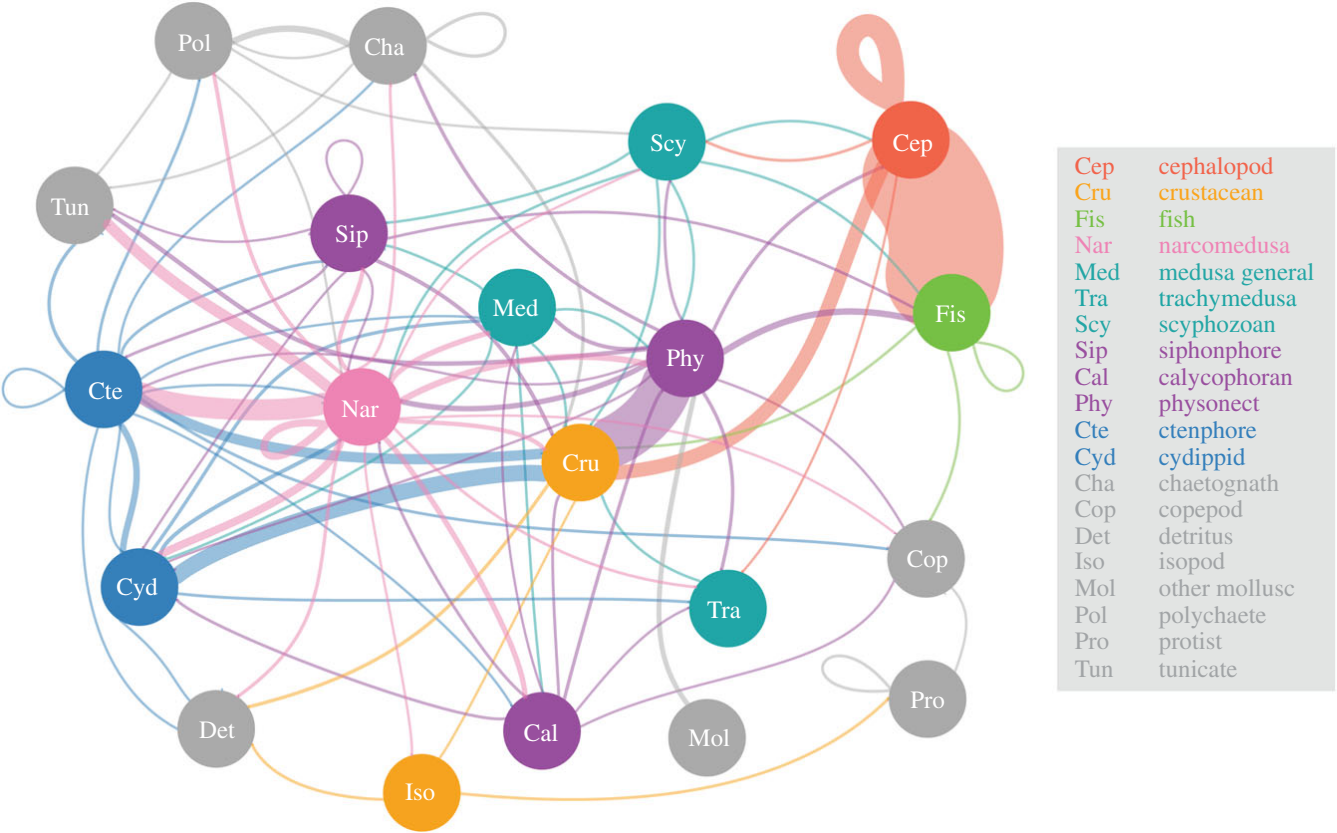
An *in situ* perspective of the food web derived from ROV-based observations of feeding, as represented by 20 broad taxonomic groupings. The linkages between predator to prey are coloured according to predator group origin, and loops indicate within-group feeding. The thickness of the lines or edges connecting food web components is scaled to the log of the number of unique ROV feeding observations across the years 1991–2016 between the two groups of animals. Absolute counts, represented here by edge thickness, are presented in figure 7. The different groups have eight colour-coded types according to main animal types as indicated by the legend and defined here: red, cephalopods; orange, crustaceans; light green, fish; dark green, medusa; purple, siphonophores; blue, ctenophores and grey, all other animals. In this plot, the vertical axis does not correspond to trophic level, because this metric is not readily estimated for all members. Note that for the *Sip* and *Med* groups, there are overlapping sub-groups (calycophoran and physonect siphonophores, and trachymedusa and scyphozoan medusa, respectively), which is attributed to varying levels of taxonomic discrimination possible from *in situ* video observations.

Tabulated predator–prey interactions were used to compile an ecosystem-level network, or food web, connected by relationships from all documented feeding events. Food webs were constructed in R (v. 3.1.2) using *igraph* and *ggplot2*. Trophic position assessments are not represented because this metric is not easily estimated for all members. Given the complexity of the predator–prey relationships and the inter-changeability of these roles, we present data according to broad ecological groups (e.g. figures 3 and 4). For some groups, more specific sub-groups are represented in tandem. This is particularly true for the ‘siphonophore’ and ‘medusa general’ groups, which are also represented by calycophoran and physonect sub-groups, and trachymedusa, narcomedusa and scyphozoan sub-groups, respectively (figure 4). This overlap occurs because all interactions are conservatively represented at the taxonomic level for which we were confident in attributing identification. Ecosystem interactions are more clearly summarized using these clustered ecological groupings, rather than groups of varying levels of taxonomic precision based on the ability to identify specimens from *in situ* observations. More detailed groupings are provided as electronic supplementary material, as well as raw data and reproducible analysis code (electronic supplementary material S1).

## 3. Results

### (a) Broad summary of feeding interactions

Seven hundred and forty-three independent predator–prey feeding interactions were identified from within the study region, between October 1991 and December 2016. Among these interactions 84 distinct types of predators and 82 separate prey concepts or categories were identified (tabulated at the most specific taxonomic identifications). Together, these feeding interactions included 242 unique combinations of predators and prey at the most specific taxonomic levels. Figure 2 depicts example frame-grab images of feeding interactions across different types of broad animal groups (fishes, crustaceans, cephalopods, siphonophores, medusae).

Twenty-five of the 743 observations lacked depth records and were thus included in the food web networks but excluded from analyses of feeding across depths. Most feeding interactions were observed in the upper 1500 m of the water column, with the deepest observation occurring at 3952 m and the shallowest at a depth of 1 m (figure 3). Across all interactions containing depth records, the median depth of observation was 401 m and the mean depth was 496 m. This part of the water column lies above the depth of the core area of the regional oxygen minimum zone. There were clear trends in the dominance of different prey types according to broad ecological groupings across the observed depths. Crustaceans were the most commonly consumed prey in the epipelagic zone, followed by fishes in both the mesopelagic and bathypelagic zones (figure 3*a*). Among predators, cephalopods were the most frequently observed across the mesopelagic and bathypelagic zones, while physonect siphonophores were the most numerically dominant predators in the epipelagic zone (figure 3*b*).

### (b) Food web structure

A synopsis of the food web of the central California deep pelagic ecosystem, as determined from 27 years of *in situ* ROV observations of feeding, is presented in figure 4. Using 20 broad ecological groupings, the most commonly observed predator–prey feeding interactions were cephalopods preying upon fishes (*n* = 230 unique feeding events), and physonect siphonophores eating crustaceans (*n* = 100). Other commonly observed feeding interactions were cephalopods feeding on other cephalopods (*n* = 42), and narcomedusae ingesting ctenophores (*n* = 39). Crustaceans serve as a central prey node (figure 4), fed upon widely by both gelatinous (siphonophores, ctenophores, hydromedusae) and non-gelatinous animals (cephalopods, fishes). Narcomedusae like *Solmissus* (figure 2*d*) and *Aegina* fed upon the highest diversity of prey groups, with predation records across 16 of the 20 different general groups presented in figure 4, pink node and pink edges. Other consumers that fed diversely included cephalopods, physonect siphonophores and ctenophores, all of which fed across 12 of the 20 prey groups. Detritivores seen feeding on ‘marine snow’ included crustaceans (mainly munnopsid isopods), and some gelatinous species (ctenophores, narcomedusae), typically considered to be carnivores. In this ecosystem, detritivores such as the polychaete *Poeobius meseres* and pseudothecosome pteropods, which gather detritus using mucus webs, are known to be abundant. While their presence is quantified in VARS, actively deployed mucus webs are not annotated as feeding events, and thus are not included in this analysis.

### (c) Seasonality in food web structure

**Figure 5.**
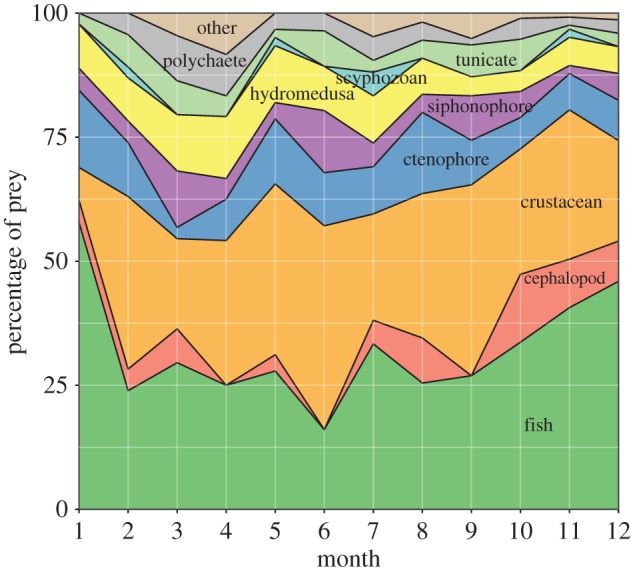
Monthly comparisons of the proportions of pelagic food web prey resources among 11 general ecological groups.

ROV feeding observations were distributed unevenly across months, limiting our ability to robustly evaluate whether food web dynamics shift seasonally. However, we did examine the relative proportions of 11 general food web components for their contribution to monthly proportions of prey resources in figure 5. A few notable differences in the relative abundances of prey types were evident between the spring and fall seasons, likely associated with the onset and cessation of regional upwelling. The importance of crustaceans and fishes decreased in March, while soft-bodied and gelatinous prey (polychaete worms, tunicates, medusae, siphonophores) were generally more important in March than during the Fall.

### (d) Changes with depth

Just as observations of feeding data were unevenly distributed across seasons (months), feeding observations were unevenly distributed throughout the 0–4000 m depth range sampled. Of the 718 observations with depth records, 8.1% were from epipelagic waters (0–200 m), 66.7% were from upper mesopelagic waters (200–600 m), 18.8% were from lower mesopelagic waters (600–1000 m) and only 6.4% were from bathypelagic waters (1000–4000 m). These depth patterns largely, but not entirely, reflect the relative amounts of ROV dive time spent within each depth interval.

For the animals with the highest number of overall observed predation events, there were clear differences in how and where in the water column feeding occurred. For example, the small physonect siphonophore *Nanomia bijuga* was observed feeding on 72 different occasions, predominantly upon euphausiids. Less frequently observed prey of *N. bijuga* included a sergestid shrimp, a narcomedusa (*Aegina*) and chaetognaths. These feeding interactions occurred between 23 and 487 m, but were concentrated around a median depth of 284 m (mean 277 m). Since 68 of the 72 predation events were on euphausiids (krill), *N. bijuga* is an example of an active, specialized predator feeding principally on one type of prey within a relatively narrow depth band [4,27]. By contrast, the large, generalist siphonophores such as *Praya dubia* and *Apolemia uvaria* (core habitat depths of 75–127 m and 575–675 m, respectively) are passive predators whose drifting tentacular curtains capture broad ranges of prey types (copepods, ctenophores, chaetognaths, narcomedusae, fishes, euphausiids), characteristic of the depths they inhabit.

**Figure 6.**
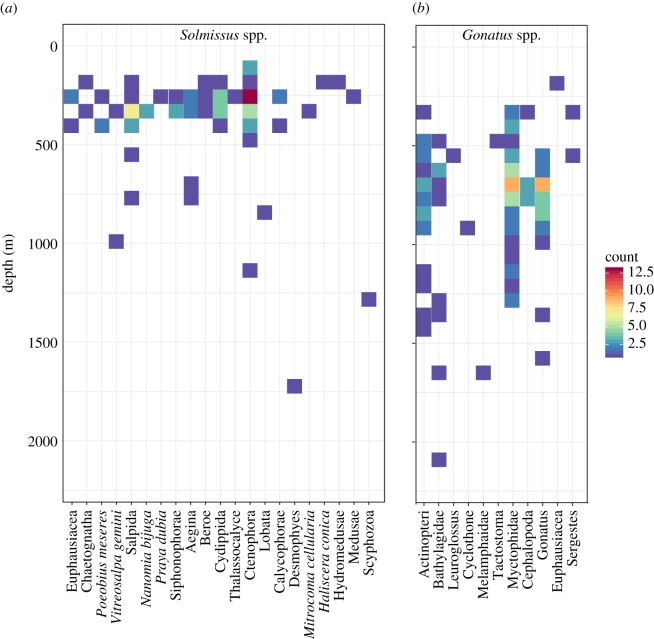
Counts and distributions of different prey types across the water column for two distinct predators: (*a*) the narcomedusae *Solmissus* spp. and (*b*) the cephalopods *Gonatus* spp. Prey types are shown as individual columns and counts of observed predation events are tabulated according to 200 m depth bins to match the colour bar scale.

Other predators fed across broad depths spanning the epipelagic and mesopelagic zones, ingesting a great diversity of prey types. The narcomedusa *Solmissus*, for example, was observed during 89 predation events at depths ranging from 94 to 1701 m, feeding on 21 different prey types (figure 6*a*). However, the majority of this feeding occurred within the 200–400 m depth band, on ctenophores, cnidarians and salps. At greater depths, the abundant squid *Gonatus* was documented feeding a total of 109 times, at depths ranging from 160 to 2057 m, on 11 different types of fishes, as well as cephalopods and crustaceans (figure 6*b*). The core of the feeding activity by *Gonatus* was centred on the depth band of 400–1000 m, on myctophid fishes and within-group cannibalism.

## 4. Discussion

**Figure 7.**
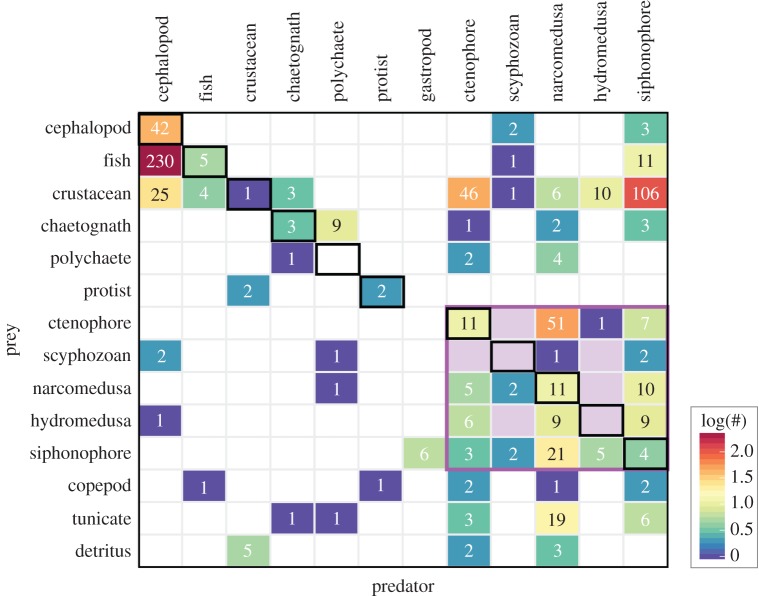
Predation matrix summarizing all observed food web interactions for the deep pelagic ecosystem of our study region, as documented by a series of ROV dives from 1991 to 2016. Broad prey groups are listed in rows down the *y*-axis, with predators shown as columns. Numbers within cells indicate total observed feeding interactions between respective predator and prey types (corresponding to the edges in figure 4), and cells are coloured according to the number of feeding interactions within a predator type. The colour scale has been log-transformed to more evenly discriminate the less frequent interactions. The ‘jelly web’ (*sensu* [4]) is highlighted with the purple box at the lower right of the matrix. Cannibalistic feeding (within the same broad group) is highlighted by black-outlined borders along the diagonal.

This study represents the first attempt to delineate the food web structure of a deep pelagic ecosystem based on *in situ* ROV observations spanning decades. Our results portray a complex food web from a different but highly complementary perspective than previous trophic investigations, which were based primarily on gut contents and biochemical markers. Direct, *in situ* observations reveal that a large proportion of the central predatory groups in midwater ecosystems are soft-bodied gelatinous animals (siphonophores, ctenophores and narcomedusae; figures 4 and 7). These organisms are rarely accounted for in other trophodynamic studies, and furthermore, are rarely identified in datasets derived from midwater trawls. The collective ecological importance and food web roles of large, complex gelatinous fauna have been previously referred to as ‘the jelly web’ [4,28]. Within this multifaceted jelly web, the distinction can be made between gelatinous animal groups that (a) feed directly on phytoplankton and sinking detritus via filter feeding or selective grazing, (b) actively hunt other gelatinous animals in addition to crustaceans and fishes and (c) passively trap or lure a wide range of prey. Here, we contrast and integrate the importance of these gelatinous animal feeding interactions with existing notions of how ecologists understand pelagic food web structure.

### (a) Primary food web pathways

In the absence of seafloor chemosynthesis, all pelagic food webs are ultimately fuelled by primary production generated in sunlit surface waters. In addition to being utilized by microbial communities, this phytoplankton-based organic matter is then directly ingested by primary consumers that are either hard-bodied zooplankton, such as copepods and krill, or by gelatinous filter feeders, such as salps and larvaceans. Conventionally, primary pathways in pelagic food webs have been qualitatively described from stomach content studies utilizing specimens captured with midwater trawl nets. Thus, the principal predators of mixed zooplankton assemblages have been typically identified as myctophid or other micronektonic fishes [29,30,31] and decapod crustaceans [32,33]. The key predators of these zooplanktivorous fishes and crustaceans include dragonfishes [34–36] and large squids [37–40]. In addition, siphonophores consume these same zooplanktivorous fishes by luring them with bioluminescent tentilla [22,41]. All together, these species comprising midwater micronekton assemblages are the forage base for many commercially important meso-predator and apex species such as marine mammals, sea birds and tunas [7,42–44]. While the predator–prey interactions of many of these midwater species are reported here in our ROV-based food web synthesis, the majority of soft-bodied and gelatinous species, which are damaged or largely missed by midwater trawling, have not been previously included in descriptions of conventional, primary pelagic food web pathways. Other feeding studies support the dominance of narcomedusae as predators of gelatinous zooplankton [45,46], and we go on here to integrate these gelatinous predator roles into broader food web understanding.

Another primary food web pathway involves sinking and suspended detritus in the form of microparticles (‘marine snow’, *sensu* [47]), and as larger detrital aggregates [48,49]. Marine snow is often directly or indirectly derived from phytoplankton, and is either filtered from the water column by salps and larvaceans, for example, or grazed upon by low-trophic level consumers such as munnopsid isopods and midwater polychaetes (e.g. *P. meseres* [50]). This detritivorous feeding guild is also known as grazers, and we show that many grazers are in turn directly consumed by narcomedusae and ctenophores, for example, forming a trophic link between organic matter and the ‘jelly web’ discussed below. As noted above, these continuously feeding detritivores are not quantitatively included in our dataset, yet would be numerically important. The link to organic detritus through grazers and their gelatinous predators can connect back to an assortment of teleost and chondrichthyan predators (reviewed by [51]). Larger detrital aggregates are also consumed by both grazers and by larger animals such as cephalopods [52], which in turn are prey for large fishes, sea birds and cetaceans.

### (b) Trophic links to the ‘jelly web’

Throughout the water column, gelatinous animals have been depicted as important predators of zooplankton (e.g. [53,54]), and yet because these same species are not frequently identified as important prey of higher-order consumers, their collective role in marine food webs has regularly been characterized as a ‘trophic dead end’ [55]. Using quantified, empirical feeding data, we demonstrate the structure and overall ecological importance of the ‘jelly web’. This importance greatly contrasts with gelatinous animals as a ‘trophic dead end’ and here, we balance their trophic roles alongside and linked to more traditional midwater taxa such as fishes, cephalopods and crustaceans, whose interactions are only a subset of the more complex picture observed with ROVs (figure 7).

A growing body of ecological evidence based on multiple trophic approaches has identified gelatinous animals as prey for higher trophic level predators, with varying degrees of importance (e.g. [51,54]). For example, multi-year diet studies of large midwater fishes such as longnose lancetfish (*Alepisaurus ferox*) and opah (*Lampris guttatus*) have documented regular consumption of salps and pyrosomes, and cephalopods that are known gelativores [7,56]. Penguins in the Southern Ocean have been observed feeding semi-regularly on carnivorous gelata [15]. The use of trophic biomarkers such as fatty acids and stable isotopes has also demonstrated clear food web links to gelatinous species (e.g. [57]). Additionally, some larger pelagic consumers such as sunfish, leatherback turtles [58], stromateoid fishes and the deep-living giant octopus [59] are known to achieve large body masses by feeding semi-exclusively on gelatinous species. Thus, combining these ecological observations with the unique ROV-based trophic observations of the midwater gelatinous assemblage presented here, the food web role(s) of gelatinous animals must be rebalanced against current pelagic food web paradigms and overall ecological understanding.

### (c) Study limitations and future work

Each method of observation or ecological tool used to gather trophic data in the midwater must be interpreted within the context of inherent limitations and biases. Commonly used midwater trawls are known to significantly underestimate the biomass of key pelagic groups depending on trawl type [60]. Additionally, diet information gathered from trawl-collected specimens can overestimate feeding rations and skew predator–prey relationships due to post-capture net feeding [8,61]. While a highly valuable source of data, diet from SCA generally provides a short-term snapshot of ingested food items, and both is sample-intensive and requires precise and detailed taxonomic expertise [6,7,43,56]. ROVs, on the other hand, while well suited for high resolution, *in situ* observations, are potentially subject to avoidance by at least some taxa that are sensitive to noise and light, and are mobile enough to escape [25]. Thus, a key limitation of an ROV-based food web biases predator and prey identities to those taxa least affected by ROV intrusiveness. However, multiple taxa that fit this characterization were continuously observed in the roles of both predator and prey. For example, cephalopods such as *Dosidicus gigas* and *Gonatus* are presented as key consumers of midwater fishes such as myctophids and bathylagids [40,62,63]. All of these animals are highly mobile and have sensitive, image-forming eyes and yet are well represented in this dataset. Future work could quantitatively weight the importance of the feeding relationships presented alongside biomass and abundance estimates for the deep pelagic community.

No single trophic assessment method should be presented as a stand-alone approach to characterizing food web structure. ROV observations are ideal for documenting the jelly web, which is difficult to assess by other methods. With mostly transparent gelatinous predators, we can see prey long after they have been captured and eaten, and ROV observations eliminate the risk of prey extrusion following sample collection. ROVs are less optimal for observation of predation by fishes and crustaceans, where already ingested prey cannot usually be visually documented without capture and dissection. While our findings are more focused on the food web roles of soft-bodied animals less sensitive to the presence of a ROV, this perspective is not generally captured by other more commonly used methods.

A second limitation of our study is the inability to separate diel feeding across depth zones, as the large majority of the observations occurred during daylight hours. Diel vertical migration is a widespread phenomenon among midwater animals, and future studies should focus on key differences between day- and night-time feeding by both migrators and non-migrators. These results do, however, demonstrate that many food web linkages are active at depth during the day, and not just in near-surface waters at night. Lastly, because the feeding observations presented were collated from over 25 years of midwater ROV programmes at MBARI, we were unable to robustly evaluate the influence of inter-annual, seasonal and depth-related variability on overall food web structure.

This study highlights the importance of a persistent and continued presence in the deep sea, demonstrating for the first time how the resultant collection of *in situ* feeding observations can be synthesized to provide both fine-scale and big-picture understanding of pelagic food webs. Integrating this quantitative feeding data into ecosystem models will support strategic resource management from a whole-ecosystem perspective [64]. Such knowledge of ecosystem function is critical for anticipating the impacts of changing environmental conditions and anthropogenic pressures such as large-scale industrialized fishing.

## Supplementary Material

R-Markdown file detailing analyses and accompanying code.

## Supplementary Material

Supplementary dataset comprising “edge” data for the food web.

## Supplementary Material

Supplementary dataset comprising “node” data for the food web.

## Supplementary Material

Expanded supplementary dataset comprising all raw feeding data utilized in this study.

## Data accessibility

All edge and node food web data are included as online electronic supplementary material. Raw data are included in S2, S3 and S4. A detailed R-Markdown file (S1) details our analyses and the incorporation of the raw data into our figures.

## References

[1] Elton C 1927 Animal ecology. London, UK: Sidgwick & Jackson.

[2] Lindeman RL 1942 The trophic-dynamic aspect of ecology. Ecology 23, 399–418. (10.2307/1930126)

[3] Libralato S, Pranovi F, Stergiou K, Link J 2014 Trophodynamics in marine ecology: 70 years after Lindeman. Mar. Ecol. Prog. Ser. 512, 1–7. (10.3354/meps11033)

[4] Robison BH 2004 Deep pelagic biology. J. Exp. Mar. Biol. Ecol. 300, 253–272. (10.1016/j.jembe.2004.01.012)

[5] Drazen JC, Sutton TT 2017 Dining in the deep: the feeding ecology of deep-sea fishes. Annu. Rev. Mar. Sci. 9, 337–366. (10.1146/annurev-marine-010816-060543) 27814034

[6] Hyslop EJ 1980 Stomach contents analysis: a review of methods and their application. J. Fish Biol. 17, 411–429. (10.1111/j.1095-8649.1980.tb02775.x)

[7] Choy CA, Portner E, Iwane M, Drazen JC 2013 Diets of five important predatory mesopelagic fishes of the central North Pacific. Mar. Ecol. Prog. Ser. 492, 169–184. (10.3354/meps10518)

[8] Lancraft T, Robison B 1980 Evidence of postcapture ingestion by midwater fishes in trawl nets. Fish. Bull. 77, 713–715.

[9] Pethybridge HR, Parrish CC, Morrongiello J, Young JW, Farley JH, Gunasekera RM, Nichols PD 2015 Spatial patterns and temperature predictions of tuna fatty acids: tracing essential nutrients and changes in primary producers. PLoS ONE 10, e0131598 (10.1371/journal.pone.0131598) 26135308 PMC4489677

[10] Choy CA, Popp BN, Hannides CCS, Drazen JC 2015 Trophic structure and food resources of epipelagic and mesopelagic fishes in the North Pacific subtropical gyre ecosystem inferred from nitrogen isotopic compositions. Limnol. Oceanogr. 60, 1156–1171. (10.1002/lno.10085)

[11] Fanelli E, Cartes JE, Papiol V 2011 Food web structure of deep-sea macrozooplankton and micronekton off the Catalan slope: insight from stable isotopes. J. Mar. Syst. 87, 79–89. (10.1016/j.jmarsys.2011.03.003)

[12] Trueman CN, Johnston G, O'Hea B, MacKenzie KM 2014 Trophic interactions of fish communities at midwater depths enhance long-term carbon storage and benthic production on continental slopes. Proc. R. Soc. B 281, 20140669 (10.1098/Rspb.2014.0669) PMC4071550 24898373

[13] Blum JD, Popp BN, Drazen JC, Choy CA, Johnson MW 2013 Evidence for methylmercury production below the mixed layer in the North Pacific Ocean. Nature Geoscience 6, 879–884. (10.1038/ngeo1918)

[14] Seminoff JA, Jones TT, Marshall GJ 2006 Underwater behaviour of green turtles monitored with video-time-depth recorders: what's missing from dive profiles? Mar. Ecol. Prog. Ser. 322, 269–280. (10.3354/meps322269)

[15] Thiebot J-B et al. 2017 Jellyfish and other gelata as food for four penguin species: insights from predator-borne videos. Front. Ecol. Environ. 15, 437–441. (10.1002/fee.1529)

[16] Pennington JT, Chavez FP 2000 Seasonal fluctuations of temperature, salinity, nitrate, chlorophyll and primary production at station H3/M1 over 1989–1996 in monterey bay, California. Deep Sea Res. Part II Top. Stud. Oceanogr. 47, 947–973. (10.1016/S0967-0645(99)00132-0)

[17] Robison BH, Sherlock RE, Reisenbichler KR 2010 The bathypelagic community of monterey canyon. Deep Sea Res. Part II Top. Stud. Oceanogr. 57, 1551–1556. (10.1016/j.dsr2.2010.02.021)

[18] Sutton TT 2013 Vertical ecology of the pelagic ocean: classical patterns and new perspectives. J. Fish Biol. 83, 1508–1527. (10.1111/Jfb.12263) 24298949

[19] Arkema KK, Abramson SC, Dewsbury BM 2006 Marine ecosystem-based management: from characterization to implementation. Front. Ecol. Environ. 4, 525–532. (10.1890/1540-9295)

[20] Doney SC et al. 2012 Climate change impacts on marine ecosystems. Annu. Rev. Mar. Sci. 4, 11–37. (10.1146/annurev-marine-041911-111611) 22457967

[21] Young JW et al. 2015 The trophodynamics of marine top predators: current knowledge, recent advances and challenges. Deep Sea Res. Part II Top. Stud. Oceanogr. 113, 170–187. (10.1016/j.dsr2.2014.05.015)

[22] Haddock SHD, Dunn CW, Pugh PR, Schnitzler CE 2005 Bioluminescent and red-fluorescent lures in a deep-sea Siphonophore. Science 309, 263 (10.1126/science.1110441) 16002609

[23] Gasca R, Suárez-Morales E, Haddock SHD 2007 Symbiotic associations between crustaceans and gelatinous zooplankton in deep and surface waters off California. Mar. Biol. 151, 233–242. (10.1007/s00227-006-0478-y)

[24] Harbison GR, Biggs DC, Madin LP 1977 The associations of *Amphipoda hyperiidea* with gelatinous zooplankton—II. Associations with Cnidaria, Ctenophora and Radiolaria. Deep Sea Res. 24, 465–488. (10.1016/0146-6291(77)90484-2)

[25] Widder EA, Robison BH, Reisenbichler KR, Haddock SHD 2005 Using red light for in situ observations of deep-sea fishes. Deep Sea Res. Part Oceanogr. Res. Pap. 52, 2077–2085. (10.1016/j.dsr.2005.06.007)

[26] Schlining BM, Stout NJ 2006 MBARI's video annotation and reference system. *OCEANS* **2006**, 1–5. (doi:10.1109/OCEANS.2006.306879)

[27] Robison BH, Reisenbichler KR, Sherlock RE, Silguero JM, Chavez FP 1998 Seasonal abundance of the siphonophore, *Nanomia bijuga*, in Monterey Bay. Deep Sea Res. Part II Top. Stud. Oceanogr. 45, 1741–1751. (10.1016/S0967-0645(98)80015-5)

[28] Purcell JE 1991 A review of Cnidarians and Ctenophores feeding on competitors in the Plankton. Hydrobiologia 216, 335–342. (10.1007/Bf00026483)

[29] Clarke TA 1973 Some aspects of the ecology of lanternfishes (Myctophidae) in the Pacific Ocean near Hawaii. Fish. Bull. 71, 401–434.

[30] Hopkins TL, Gartner JV 1992 Resource-partitioning and predation impact of a low-latitude myctophid community. Mar. Biol. 114, 185–197. (10.1007/BF00349518)

[31] Cailliet GM, Ebeling AW 1990 The vertical distribution and feeding habits of two common midwater fishes (*Leuroglossus* *stilbius* and *Stenobrachius leucopsaras*) off Santa Barbara. Calif. Coop. Ocean. Fish. Investig. Rep. 31, 106–123.

[32] Hopkins TL, Flock ME, Gartner JV, Torres JJ 1994 Structure and trophic ecology of a low latitude midwater decapod and mysid assemblage. Mar. Ecol. Prog. Ser. 109, 143 (10.3354/Meps109143)

[33] Walters JF 1976 Ecology of Hawaiian sergestid shrimps (Penaeidea: Sergestidae). Fish Bull. 74, 799–836.

[34] Clarke TA 1982 Feeding habits of Stomiatoid fishes from Hawaiian waters. Fish. Bull. 80, 287–304.

[35] Sutton TT, Hopkins TL 1996 Trophic ecology of the stomiid (Pisces: Stomiidae) fish assemblage of the eastern Gulf of Mexico: strategies, selectivity and impact of a top mesopelagic predator group. Mar. Biol. 127, 179–192. (10.1007/Bf00942102)

[36] Fisher JP, Pearcy WG 1983 Reproduction, growth and feeding of the mesopelagic fish *Tactostoma macropus* (Melanostomiatidae). Mar. Biol. 74, 257–267. (10.1007/BF00403449)

[37] Cherel Y, Ridoux V, Spitz J, Richard P 2009 Stable isotopes document the trophic structure of a deep-sea cephalopod assemblage including giant octopod and giant squid. Biol. Lett. 5, 364–367. (10.1098/rsbl.2009.0024) 19324634 PMC2679927

[38] Parry M 2006 Feeding behavior of two ommastrephid squids, *Ommastrephes bartramii* and *Sthenoteuthis oualaniensis* off Hawaii. Mar. Ecol. Prog. Ser. 318, 229–235. (10.3354/meps318229)

[39] Passarella KC, Hopkins TL 1991 Species composition and food habits of the micronektonic cephalopod assemblage in the eastern Gulf of Mexico. Bull. Mar. Sci. 49, 638–659.

[40] Field JC et al. 2013 Foraging ecology and movement patterns of jumbo squid (*Dosidicus gigas*) in the California Current System. Deep Sea Res. Part II Top. Stud. Oceanogr. 95, 37–51. (10.1016/j.dsr2.2012.09.006)

[41] Pugh PR, Haddock SHD 2016 A description of two new species of the genus *Erenna* (Siphonophora: Physonectae: Erennidae), with notes on recently collected specimens of other Erenna species. Zootaxa 4189, 401 (10.11646/zootaxa.4189.3.1) 27988743

[42] Harrison CS, Seki MP 1987 Trophic relationships among tropical seabirds at the Hawaiian Islands. In Seabirds: feeding ecology and role in marine ecosystems (ed. Croxall JP), pp. 305–326. Cambridge, UK: Cambridge University Press.

[43] Duffy LM et al. 2017 Global trophic ecology of yellowfin, bigeye, and albacore tunas: understanding predation on micronekton communities at ocean-basin scales. Deep Sea Res. Part II Top. Stud. Oceanogr. 140, 55–73. (10.1016/j.dsr2.2017.03.003)

[44] Pauly D, Trites AW, Capuli E, Christensen V 1998 Diet composition and trophic levels of marine mammals. ICES J. Mar. Sci. 55, 467–481. (10.1006/jmsc.1997.0280)

[45] Raskoff KA 2002 Foraging, prey capture, and gut contents of the mesopelagic narcomedusa *Solmissus* spp. (Cnidaria: Hydrozoa). Mar. Biol. 141, 1099–1107. (10.1007/s00227-002-0912-8)

[46] Mills CE, Goy J 1988 In situ observations of the behavior of mesopelagic *Solmissus* Narcomedusae (Cnidaria, Hydrozoa). Bull. Mar. Sci. 43, 739–751.

[47] Alldredge AL, Silver MW 1988 Characteristics, dynamics and significance of marine snow. Prog. Oceanogr. 20, 41–82. (10.1016/0079-6611(88)90053-5)

[48] Silver MW, Coale SL, Pilskaln CH, Steinberg DR 1998 Giant aggregates: importance as microbial centers and agents of material flux in the mesopelagic zone. Limnol. Oceanogr. 43, 498–507. (10.4319/lo.1998.43.3.0498)

[49] Robison BH, Reisenbichler KR, Sherlock RE 2005 Giant larvacean houses: rapid carbon transport to the deep sea floor. Science 308, 1609–1611. (10.1126/science.1109104) 15947183

[50] Uttal L, Buck KR 1996 Dietary study of the midwater polychaete *Poeobius meseres* in Monterey Bay, California. Mar. Biol. 125, 333–343. (10.1007/BF00346314)

[51] Arai MN 2005 Predation on pelagic coelenterates: a review. J. Mar. Biol. Assoc. U. K. 85, 523–536. (10.1017/S0025315405011458)

[52] Villanueva R, Perricone V, Fiorito G. 2017 Cephalopods as predators: a short journey among behavioral flexibilities, adaptions, and feeding habits. Front. Physiol. 8, 598 (10.3389/fphys.2017.00598) 28861006 PMC5563153

[53] Purcell JE 1981 Dietary composition and diel feeding patterns of epipelagic siphonophores. Mar. Biol. 65, 83–90. (10.1007/BF00397071)

[54] Pauly D, Graham W, Libralato S, Morissette L, Palomares MLD 2009 Jellyfish in ecosystems, online databases, and ecosystem models. Hydrobiologia 616, 67–85. (10.1007/s10750-008-9583-x)

[55] Verity PG, Smetacek V 1996 Organism life cycles, predation, and the structure of marine pelagic ecosystems. Mar. Ecol. Prog. Ser. 130, 277–293. (10.3354/meps130277)

[56] Portner EJ, Polovina JJ, Choy CA 2017 Patterns in micronekton diversity across the North Pacific subtropical gyre observed from the diet of longnose lancetfish (*Alepisaurus ferox*). Deep Sea Res. Part Oceanogr. Res. Pap. 125, 40–51. (10.1016/j.dsr.2017.04.013)

[57] Pitt KA, Connolly RM, Meziane T 2009 Stable isotope and fatty acid tracers in energy and nutrient studies of jellyfish: a review. Hydrobiologia 616, 119–132. (10.1007/s10750-008-9581-z)

[58] Houghton JD, Doyle TK, Wilson MW, Davenport J, Hays GC 2006 Jellyfish aggregations and leatherback turtle foraging patterns in a temperate coastal environment. Ecology 87, 1967–1972. (10.1890/0012-9658(2006)87%5B1967:JAALTF%5D2.0.CO;2) 16937635

[59] Hoving HJT, Haddock SHD 2017 The giant deep-sea octopus *Haliphron atlanticus* forages on gelatinous fauna. Sci. Rep. 7, 44952 (10.1038/srep44952) 28344325 PMC5366804

[60] Kaartvedt S, Staby A, Aksnes DL 2012 Efficient trawl avoidance by mesopelagic fishes causes large underestimation of their biomass. Mar. Ecol. Prog. Ser. 456, 1–6. (10.3354/Meps09785)

[61] Clarke TA 1978 Diel feeding patterns of 16 species of mesopelagic fishes from Hawaiian waters. Fish. Bull. 76, 495–513.

[62] Hoving HJT, Robison BH 2016 Deep-sea in situ observations of gonatid squid and their prey reveal high occurrence of cannibalism. Deep Sea Res. Part Oceanogr. Res. Pap. 116, 94–98. (10.1016/j.dsr.2016.08.001)

[63] Zeidberg LD, Robison BH 2007 Invasive range expansion by the Humboldt squid, *Dosidicus gigas*, in the eastern North Pacific. Proc. Natl Acad. Sci. USA 104, 12 948–12 950. (10.1073/pnas.0702043104) PMC1937572 17646649

[64] Pethybridge HR, Choy CA, Polovina JJ, Fulton EA In press Improving marine ecosystem models with biochemical tracers. Annu. Rev. Mar. Sci. (10.1146/annurev-marine-121916-063256) 29298140

