# Supplementary material for: Deep pelagic food web structure as revealed by in situ feeding observations
Source: Proc Biol Sci. 2017 Dec 6;284(1868):20172116. doi: 10.1098/rspb.2017.2116 (PMC5740285; doi:10.1098/rspb.2017.2116)

Deep pelagic food web structure as revealed by in situ feeding observations

C. Anela Choy, Steven H. D. Haddock, Bruce H. Robison

October 2017

VARS Food web plots and data

This is an R Markdown document, provided to reproduce the data analysis and figures presented in manuscript "Deep pelagic food web structure as revealed by in situ feeding observations" by C. Anela Choy (anela@mbari.org), Steven H.D. Haddock (haddock@mbari.org), and Bruce H. Robison (robr@mbari.org), as published in Proceedings of the Royal Society B.

The primary datasets used in this study are also made available with the submitted manuscript, and linked to analysis through this R Markdown document.

Requires libraries: ggplot2, RColorBrewer, igraph, ggraph

Network graphs

Create a feeding network plot with the links colored by *predator* type, and the width by the number of interactions.

```
# don't want it to change each time
set.seed(18)

l <- layout.graphopt(net)
l <- norm_coords(l, ymin=-1, ymax=1, xmin=-1, xmax=1)

E(net)$edge.color =  adjustcolor(colorvalues[E(net)$from_group],.6)

plot.igraph(net,edge.arrow.size=0.03, edge.color=E(net)$edge.color,
                        edge.arrow.mode=2, rescale=TRUE, layout=l, edge.curved=.4,
                        vertex.label.color= "white",vertex.label.family="sans")
```

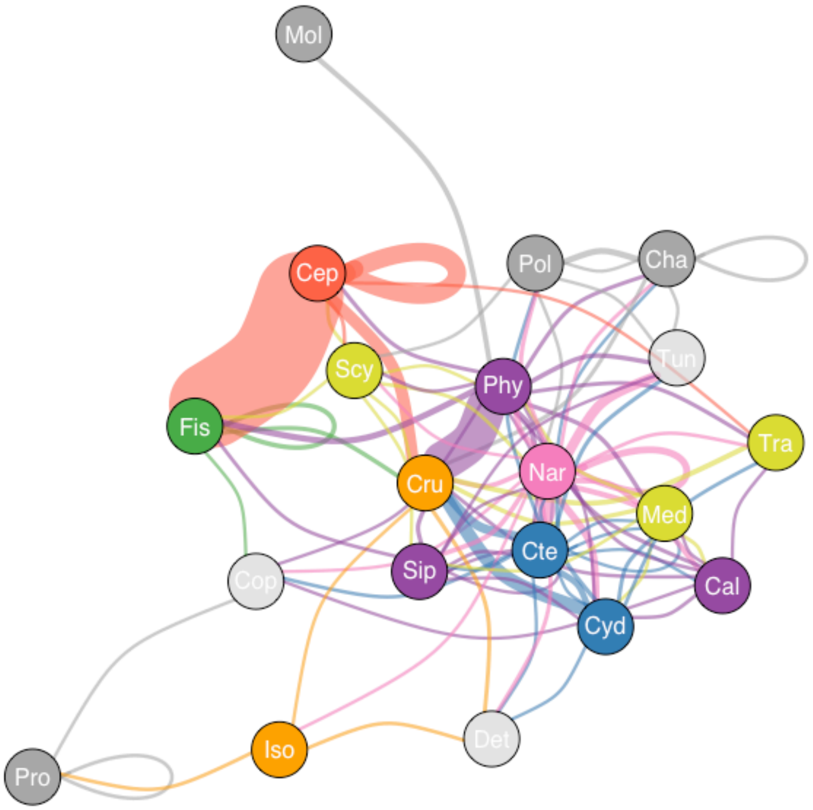


```
# color by prey
```

Create another network plot with the links colored by *prey* type.

```
E(net)$edge.color =  adjustcolor(colorvalues[E(net)$to_group],.6)
plot.igraph(net,edge.arrow.size=0.03, edge.color=E(net)$edge.color,
                              edge.arrow.mode=2, rescale=TRUE, layout=l, edge.curved=.4,
                              vertex.label.color= "white",vertex.label.family="sans")

legend(x=0, y=-1.1, names(colorvalues),pch=21, col='#000000',
       pt.bg=colorvalues, pt.cex=2, cex=.7, bty='n', ncol=3)
```

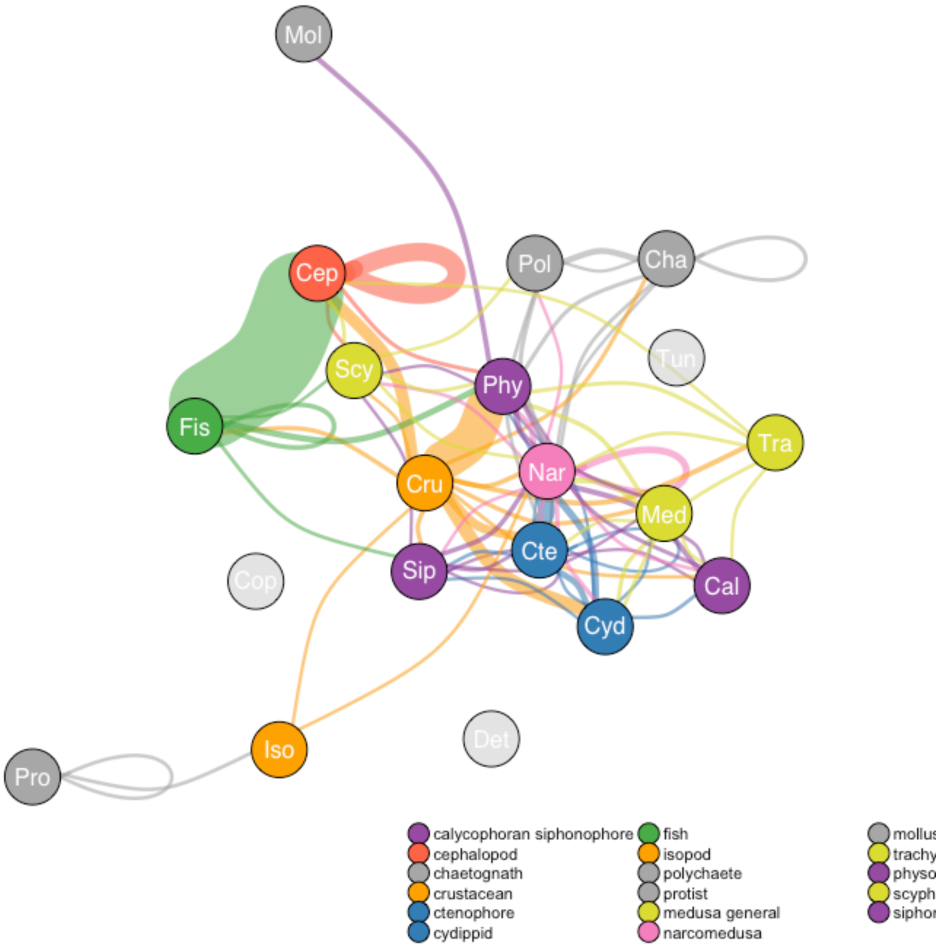

Feeding relationship matrix / heatmap

Show a matrix with predators in columns and their prey in rows. Numbers in boxes indicate number of feeding interactions for that pair.

Make a plot of the matrix.

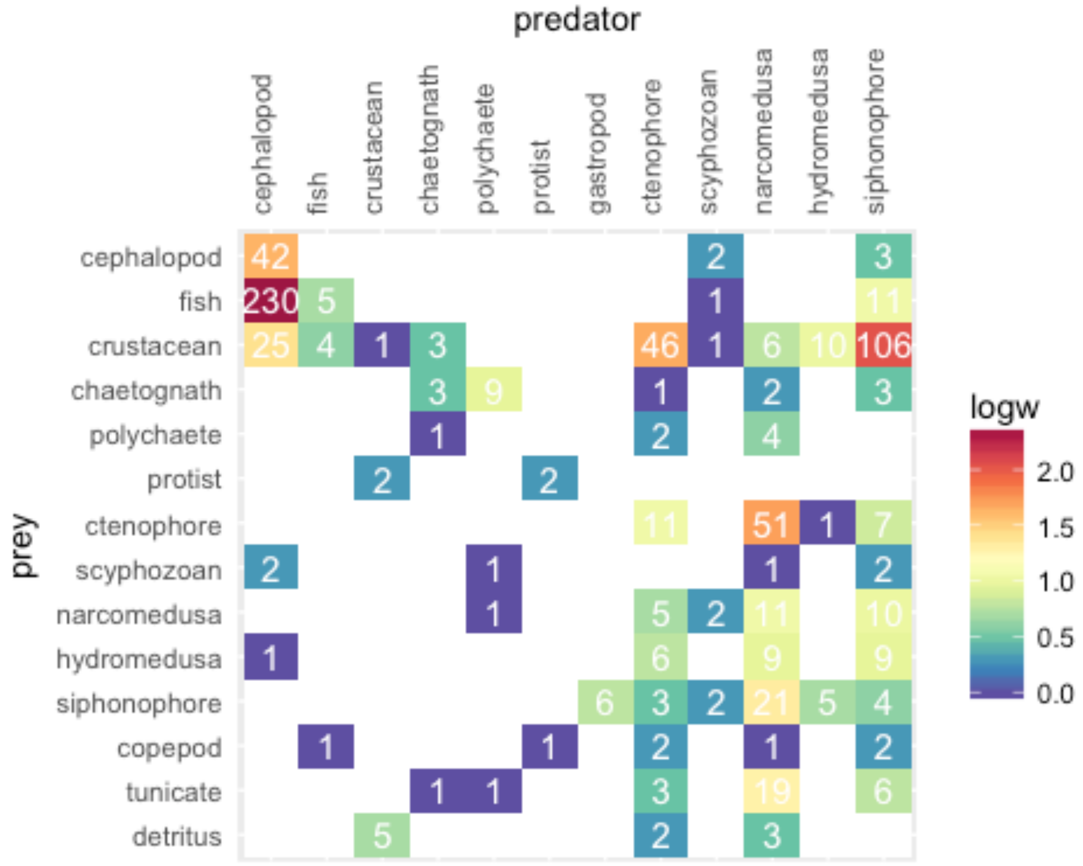

Supplement: R-Markdown file detailing analyses and accompanying code. [file rspb20172116supp1.pdf]
